# Supplementary material for: Causal Relationship between Aging and Anorexia Nervosa: A White-Matter-Microstructure-Mediated Mendelian Randomization Analysis
Source: Biomedicines. 2024 Aug 16;12(8):1874. doi: 10.3390/biomedicines12081874 (PMC11351342; doi:10.3390/biomedicines12081874)
Supplement: Supplementary file 1 [file biomedicines-12-01874-s001.zip › Supplementary Figure/Figure S4/Singe SNP plot of MR analysis for TL to ubm-b-1574.pdf]

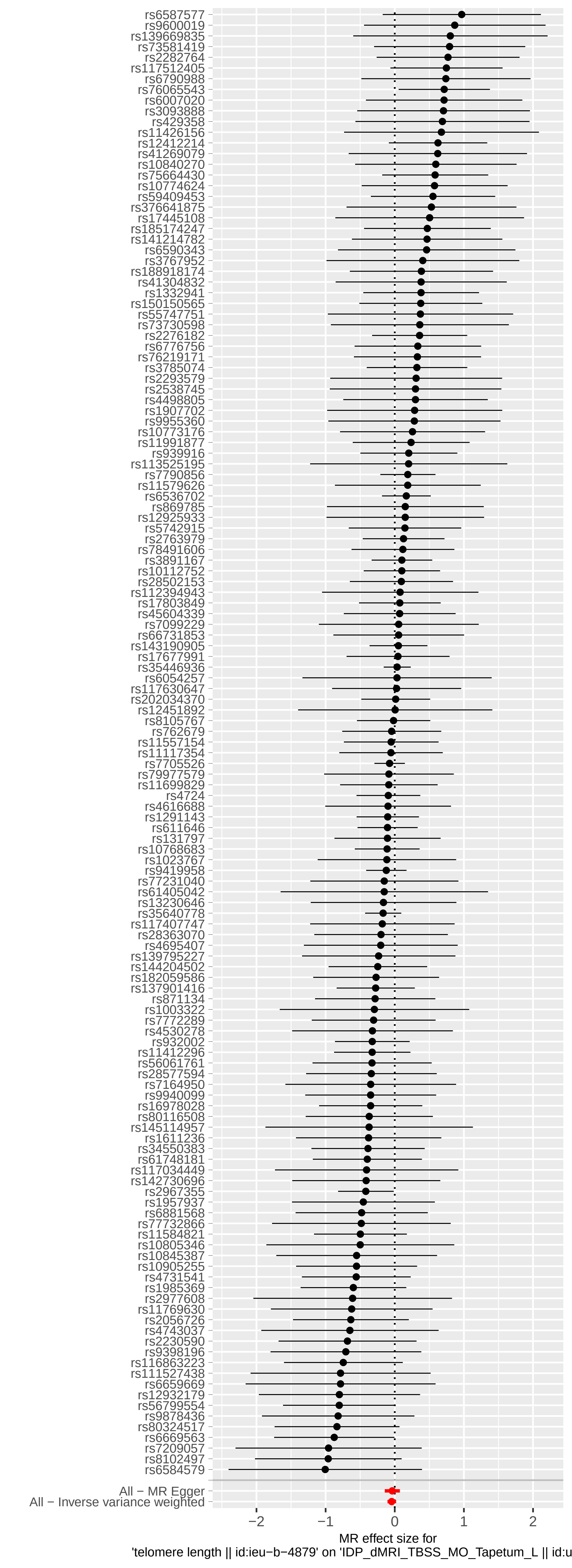
rs6587577
rs9600019
rs139669835
rs73581419
rs2282764
rs117512405
rs6790988
rs76065543
rs6007020
rs3093888
rs429358
rs11426156
rs12412214
rs41269079
rs10840270
rs75664430
rs10774624
rs59409453
rs376641875
rs17445108
rs185174247
rs141214782
rs6590343
rs3767952
rs188918174
rs41304832
rs1332941
rs150150565
rs55747751
rs73730598
rs2276182
rs6776756
rs76219171
rs3785074
rs2293579
rs2538745
rs4498805
rs1907702
rs9955360
rs10773176
rs11991877
rs939916
rs113525195
rs7790856
rs11579626
rs6536702
rs869785
rs12925933
rs5742915
rs2763979
rs78491606
rs3891167
rs10112752
rs28502153
rs112394943
rs17803849
rs45604339
rs7099229
rs66731853
rs143190905
rs17677991
rs35446936
rs6054257
rs117630647
rs202034370
rs12451892
rs8105767
rs762679
rs11557154
rs11117354
rs7705526
rs79977579
rs11699829
rs4724
rs4616688
rs1291143
rs611646
rs131797
rs10768683
rs1023767
rs9419958
rs77231040
rs61405042
rs13230646
rs35640778
rs117407747
rs28363070
rs4695407
rs139795227
rs144204502
rs182059586
rs137901416
rs871134
rs1003322
rs7772289
rs4530278
rs932002
rs11412296
rs56061761
rs28577594
rs7164950
rs9940099
rs16978028
rs80116508
rs145114957
rs1611236
rs34550383
rs61748181
rs117034449
rs142730696
rs2967355
rs1957937
rs6881568
rs77732866
rs11584821
rs10805346
rs10845387
rs10905255
rs4731541
rs1985369
rs2977608
rs11769630
rs2056726
rs4743037
rs2230590
rs9398196
rs116863223
rs111527438
rs6659669
rs12932179
rs56799554
rs9878436
rs80324517
rs6669563
rs7209057
rs8102497
rs6584579
All − MR Egger
All − Inverse variance weighted
−2
−1
0
1
2
MR effect size for
'telomere length || id:ieu−b−4879' on 'IDP_dMRI_TBSS_MO_Tapetum_L || id:u
